# Supplementary material for: Identification of novel Plasmodium vivax proteins associated with protection against clinical malaria
Source: Front Cell Infect Microbiol. 2023 Jan 25;13:1076150. doi: 10.3389/fcimb.2023.1076150 (PMC9905245; doi:10.3389/fcimb.2023.1076150)
Supplement: Supplementary file 3 [file Presentation_1.pdf]

Supplementary Material

1 Supplementary Data

Supplementary Data 1: AlphaScreen data.

Supplementary Data 2: Epidemiological data.

2 Supplementary Figures and Tables

2.1 Supplementary Figures

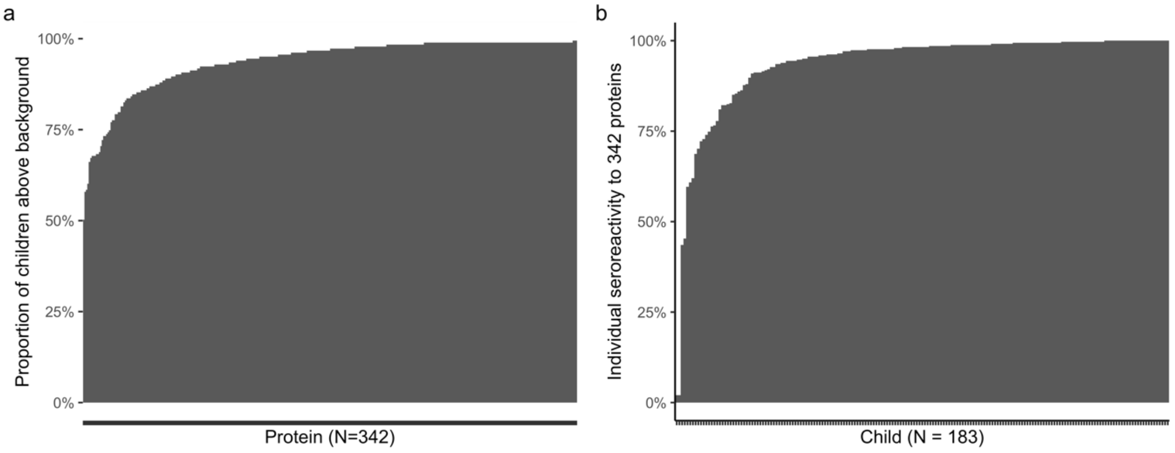

Supplementary Figure 1. Seroreactivity of the 342 *P. vivax* proteins in Papua New Guinean children. A) The proportion of children with IgG levels above the defined background (half the lowest non-negative value for each protein). All proteins had more than 50% of children above background. B) Variation in individual seroreactivity. Data shown is the proportion of the protein panel (n=342 proteins) each child is seroreactive against.

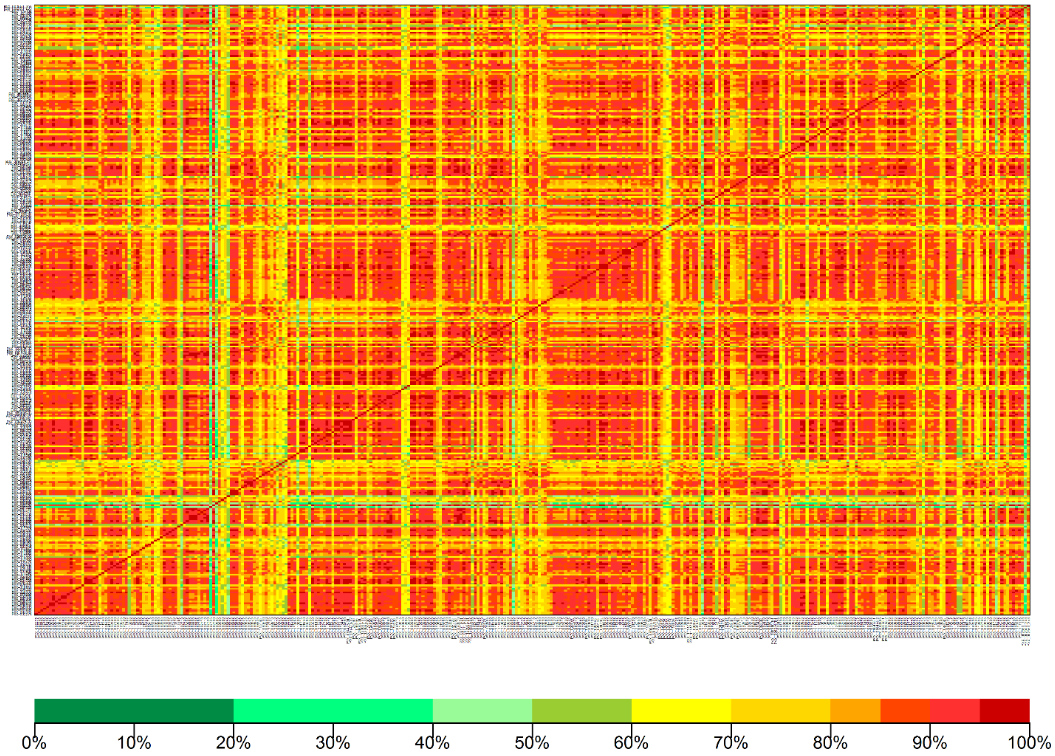

Supplementary Figure 2. Correlation between IgG measurements to 342 *P. vivax* proteins. Pairwise correlations of IgG Antibody Units between all 342 *P. vivax* antigens.
